# Treatment of *Actinomyces europaeus* and *Clostridium innocuum* necrotizing fasciitis: Case report and literature review

**DOI:** 10.1093/ajhp/zxaf190

**Published:** 2025-07-23

**Authors:** Lisa Avery, Julia Kufel, Russell Rawlings

**Affiliations:** Pharmacy Department, St. Joseph’s Health, Syracuse, NY; Department of Pharmacy Practice, St. John Fisher University, Wegmans School of Pharmacy, Rochester, NY, USA; Department of Pharmacy Practice, St. John Fisher University, Wegmans School of Pharmacy, Rochester, NY, USA; Laboratory Alliance of Central New York, Syracuse, NY, USA

**Keywords:** *Actinomyces*, *Clostridium*, necrotizing fasciitis

## Abstract

**Purpose:**

*Actinomyces europaeus* and *Clostridium innocuum* are rare causes of necrotizing fasciitis. For both organisms, it is necessary to know the specific species because variations in antibiotic sensitivity exist, making treatment challenging.

**Summary:**

An 86-year-old white female presented to the emergency department with severe pain in her right thigh. A computed tomography scan showed pockets of gas and edema in the subcutaneous tissue extending from the right lateral hemipelvis to the right inguinal area and proximal thigh. Her LRINEC (Laboratory Risk Indicator for Necrotizing Fasciitis) score was 7. The patient was started on intravenous vancomycin, piperacillin/tazobactam, and clindamycin and brought to the operating room for debridement. Anaerobic cultures grew *Bacteroides fragilis*, *Bacteroides uniformis*, and *C. innocuum*. Aerobic cultures were positive for *Actinomyces*. The laboratory was called to determine the species of *Actinomyces* and reported *A. europaeus*. The patient’s antibiotics were tailored to meropenem 2 g intravenously every 8 hours and linezolid 600 mg intravenously every 12 hours. The organisms were sent to a reference laboratory for sensitivity testing. Interpretation of sensitivity data and treatment recommendations including duration of therapy are discussed.

**Conclusion:**

It is important to rapidly identify the species for both *Actinomyces* and *Clostridium* infections and obtain sensitivity values to help ensure proper therapy is given. Interpretation of minimum inhibitory concentrations is difficult without established breakpoints. Piperacillin/tazobactam resistance has been reported for *A. europaeus*, while vancomycin resistance has been reported for *C. innocuum*. For *Actinomyces* infections, duration of therapy should be individualized and can range from 14 days to 1 year based on patient response and the species.

Key Points

- For patients with an *Actinomyces* or *Clostridium* infection, it is imperative to have the microbiology laboratory report the species identified.
- Depending on the species of *Actinomyces* and *Clostridium* identified, there may be significant differences in antibiotic susceptibility.
- Both *Actinomyces europaeus* and *Clostridium innocuum* are more resistant to antibiotics than other members of each genus, so susceptibility testing is important for these organisms.

Necrotizing fasciitis is a life-threatening skin and soft tissue infection with a prevalence of 0.4 to 7.7 cases per 100,000 people.^1^ Even with proper treatment, mortality rates range from 10% to 15%.^2^ It is commonly caused by *Streptococcus pyogenes* or *Staphylococcus aureus* or a combination of anaerobic organisms and gram-negative pathogens. The combination of *Actinomyces* and *Clostridium* species is a rare cause. *Actinomyces* species are slow-growing gram-positive facultative anaerobic bacilli. As a fastidious organism, *Actinomyces* species have very complicated nutritional requirements, making it difficult to identify them in the microbiology laboratory given that they typically grow very slowly. The most common species associated with human disease include *Actinomyces israelii*, *Actinomyces europaeus* (renamed *Gleimia europaea*), *Actinomyces meyeri* (renamed *Schaalia meyeri*), *Actinomyces odontolyticus* (renamed *Schaalia odontolytica*), *Actinomyces viscosus*, and *Actinomyces naeslundii*. *Clostridium* species are obligate anaerobic gram-positive rods that are members of the Firmicutes phylum. There are multiple species of *Clostridium*, including *Clostridium perfringens* and *Clostridium tetani*. Members of the RIC group of *Clostridium*, which comprises *Clostridium ramosum*, *Clostridium innocuum*, and *Clostridium clostridioforme*, are routinely misidentified due to Gram stain variability, rare formation of spores, atypical morphology, and variable antibiotic susceptibility. *Actinomyces* and *Clostridium* species inhabit the gastrointestinal tract, and *Actinomyces* species are also present on mucosal surfaces such as the oral cavity, urinary tract, female genital tract, and skin. Although neither is generally considered a virulent organism, their pathogenicity is enhanced in the presence of other anaerobic pathogens, such as *Bacteroides* spcies.^3^ In cases where the mucosal barrier is disrupted, such as in trauma, human bites, surgery, dental manipulations, mucositis, or insertion of an intrauterine contraceptive device, *Actinomyces* species can cause an active infection. Infection most commonly occurs at cervicofacial sites, which account for 40% to 60% of cases, and cutaneous infections represent only 3% to 5% of cases.^4^ Skin and soft tissue infections range from superficial cutaneous abscesses to necrotizing fasciitis. *C. innocuum* is a commensal organism of the gastrointestinal tract and is most often associated with diarrheal infections. It has also been reported to cause bacteremia, endocarditis, osteomyelitis, and peritonitis, but not skin infections.^5-8^

This case report reviews lessons learned when caring for a patient with necrotizing fasciitis caused by *A. europaeus*, *C. innocuum*, and *Bacteroides* species. Treatment recommendations for *Actinomyces* were established in the 1960s, with limited literature published in subsequent years.^9^ No cases of *C. innocuum* necrotizing fasciitis have been reported. Susceptibility testing, interpretation, resistance patterns, and treatment recommendations are reviewed.

## Case report

An 86-year-old white female with a past medical history significant for hypothyroidism, diabetes mellitus, glaucoma, hypertension, and hyperlipidemia presented to the emergency department for pain in her right thigh. She stated that she had noticed a bump on her right thigh about 2 weeks ago, which progressively increased in size and became more painful. Because the pain was so severe, she had been unable to stand for the past week.

The patient’s vitals on admission included a temperature of 36.8 °C (98.2 °F), heart rate of 108 beats per minute, respiratory rate of 20 breaths per minute, blood pressure of 111/77 mm Hg, and pulse oximetry reading of 98% on room air. On physical examination, the right medial thigh was found to be swollen, erythematous, and indurated extending toward the right hip and the labia, but the vaginal region did not appear to be involved. No skin openings or drainage was seen. The patient was obese, having undergone gastric bypass surgery 15 years ago, with a weight of 112 kg and body mass index of 43.7 kg/m^2^. A computed tomography scan without contrast showed multiple pockets of gas and edema in the subcutaneous tissue extending from the right lateral hemipelvis to the right inguinal area and proximal thigh. Some gas was apparent in the fascia but did not extend into the muscle layer. In laboratory testing, the patient was found to have leukocytosis, with a leukocyte concentration of 20.1 × 10^3^/μL, and had a hemoglobin concentration of 13.9 g/dL, sodium concentration of 127 mEq/L, glucose concentration of 7,712 mg/dL, blood urea nitrogen/serum creatinine ratio of 43.5, creatine kinase concentration of 16 U/L, lactate concentration of 18.0 mg/dL, and glycated hemoglobin level of 9.6 %. The patient’s LRINEC (Laboratory Risk Indicator for Necrotizing Fasciitis) score was 7, although a C-reactive protein concentration was not obtained. She was started on vancomycin 2 g intravenously (IV), piperacillin/tazobactam 4.5 g IV over 30 minutes, and clindamycin 900 mg IV. The patient was immediately brought to the operating room for incision, drainage, and debridement of the area. When the area was opened, foul-smelling purulent material was immediately apparent, which was sent for aerobic, anaerobic, and fungal cultures. The debrided area measured 50 cm in length. Postoperatively, the patient was transferred to the intensive care unit, where she was started on vasopressors and continued to receive piperacillin/tazobactam 4.5 g IV over 4 hours every 8 hours, clindamycin 600 mg IV every 8 hours, and vancomycin IV to be dosed according to measured levels.

The patient’s right thigh anaerobic cultures grew *Bacteroides fragilis*, *Bacteroides uniformis*, and *C. innocuum*. The aerobic wound culture had a few gram-positive cocci and a few gram-negative rods and was positive for *Actinomyces* species. The laboratory was asked to determine the *Actinomyces* species, which was reported as *A. europaeus*. The patient’s antibiotics were tailored to meropenem 2 g IV every 8 hours and linezolid 600 mg IV every 12 hours. Organisms were sent to a reference laboratory for sensitivity testing.

The patient returned to the operating room on days 3, 4, 5, and 7 for further debridement. On day 7, there was no further tracking or tunneling and a vacuum-assisted closure device was placed. On day 8, the patient was transferred out of the intensive care unit. She returned to the operating room on days 10, 12, and 14 for further washout and wound vacuum exchange to ensure the margins remained viable. There were no signs of further pus pockets, tracking, or tunneling. No new cultures were obtained given that the causative organisms had previously been identified and the patient was believed to be on an appropriate antimicrobial regimen. Antimicrobial sensitivity results were returned on day 15 for *C. innocuum* and *B. fragilis* (Table 1). *A. europaeus* sensitivity testing was not performed because the laboratory could not isolate the organism for testing. The rehabilitation plan was extensively discussed, and the patient chose not to undergo any additional restorative care, because she knew she would not be able to return to her baseline level of health. After 14 days of therapy with meropenem and linezolid, she was transitioned to comfort care and died on hospital day 30.

**Table 1.** Organisms Susceptibility Results

Antibiotic	*Bacteroides fragilis*		*Clostridium innocuum*	
	MIC	Interpretation	MIC	Interpretation
Ampicillin/sulbactam	1/0.5 mg/L	Susceptible	<0.25/0.12 mg/L	No interpretation
β-lactamase	Not reported	Not reported	Negative	Negative
Clindamycin	1 mg/L	Susceptible	1 mg/L	No interpretation
Meropenem	0.12 mg/L	Susceptible	2 mg/L	No interpretation
Metronidazole	1 mg/L	Susceptible	2 mg/L	No interpretation
Penicillin	16 mg/L	Resistant	<0.5 mg/L	No interpretation
Piperacillin/tazobactam	<1/4 mg/L	Susceptible	<1/4 mg/L	No interpretation

Abbreviation: MIC, minimum inhibitory concentration.

## Discussion

IV vancomycin, piperacillin/tazobactam, and clindamycin were started empirically in this patient. Interestingly, vancomycin and clindamycin would not have provided adequate empiric coverage for C. *innocuum* and piperacillin/tazobactam and clindamycin may not have been adequate for *A. europaeus*. Clindamycin was discontinued after 72 hours because *A. europaeus* and *C. innocuum* do not produce endotoxin. Piperacillin/tazobactam was changed to IV meropenem on day 5 due to the risk of piperacillin/tazobactam resistance. Ampicillin/sulbactam was recommended, but the infectious diseases consult team wanted to continue broad-spectrum coverage because gram-negative organisms were present on the aerobic wound culture. Linezolid was empirically continued throughout the treatment course, due to the severity of the infection and to provide coverage against *C. innocuum*. The patient responded to the aggressive surgical debridement and antibiotic therapy. Unfortunately, in this case, the patient chose to withdraw from therapy, so consideration of oral therapy and total duration was unnecessary.

Unlike infections with other cutaneous *Actinomyces* species, necrotizing infections present with acute, severe pain and swelling at the site of infection accompanied by systemic signs and symptoms of infection. In some cases, patients report a previous abscess at the site of infection. Our patient noticed a lesion on her leg 2 weeks before presenting to the emergency department and developed excruciating pain that immobilized her. The most common site of infection is the groin and perineum area, although infections can also occur at the site of illicit drug administration. A total of 17 cases of *Actinomyces* necrotizing fasciitis have been reported (Table 2).^10-26^ Identified species causing necrotizing fasciitis included *A. europaeus* (n = 4), A. *turicensis* (n = 4), *A. meyeri* (n = 2), *A*. *odontolytica* (n = 2), and *A. naeslundii* (n = 1). Most of the cases reported were polymicrobial, similar to our case. In 11 cases, patients were coinfected by gram-positive and gram-negative aerobes or anaerobes. None of the reported cases identified *C. innocuum* as a co-pathogen.

**Table 2.** Cases of Actinomyces Necrotizing Fasciitis

Report	Site of infection	*Actinomyces* species	Coinfecting organism(s)	Treatment regimen	Duration of therapy	Outcome (time of follow-up)
Kus et al^10^	Right proximal lower extremity, perineum, inguinal region, ischiorectal fossa	*A. europaeus*	*Actinotignum schaalii*	Empiric: piperacillin/tazobactam, vancomycin, and clindamycin Definitive: NR Oral: amoxicillin/clavulanate and metronidazole	NR	Cured (NR)
Anthony et al^11^	Lower abdomen, flank, medial thigh, labia, buttocks	*A. europaeus*	*Peptostreptococcus* spp.	Empiric: clindamycin, meropenem, and vancomycin Definitive: piperacillin/tazobactam followed by vancomycin, meropenem, levofloxacin, and micafungin given clinical worsening Oral: NR	NR	Died (day 12)
Allen et al^12^	Groin, flank, lower anterior abdominal wall	*A. europaeus*	None	Empiric: flucloxacillin Definitive: linezolid and ciprofloxacin followed by meropenem, metronidazole, and linezolid given clinical worsening Oral: NR	IV: 7 days; oral: 14 days	Cured (NR)
Zhang et al^13^	Fournier’s gangrene	*A. europaeus*	None	Empiric: cefoperazone/sulbactam and levofloxacin Definitive: piperacillin/tazobactam Oral: NR	NR	NR
Mao et al^14^	Fournier’s gangrene	*A. turicensis*	None	Empiric: cefoperazone/sulbactam Definitive: piperacillin/tazobactam and clindamycin Oral: NR	NR	Cured (3 months)
Khan et al^15^	Fournier’s gangrene	*A. turicensis*	*Streptococcus anginosus*, *Peptoniphilus harei*	Empiric: piperacillin/tazobactam, vancomycin, and clindamycin Definitive: ampicillin/sulbactam Oral: amoxicillin/clavulanate 875 mg/125 mg twice daily	IV/oral: 21 days total	Cured (18 days)
Kuzaka et al^16^	Fournier’s gangrene	*A. naeslundii*	NR	Empiric: ampicillin or third-generation cephalosporin and metronidazole ± aminoglycoside Definitive: NR Oral: NR	NR	NR
Goldberg et al^17^	Gluteus maximus	NR	*Gardnerella vaginalis*, *Peptostreptococcus* spp., *Prevotella melaninogenica*, *Prevotella loescheii*	Empiric: piperacillin/tazobactam Definitive: NR Oral: NR	IV/oral: 43 days total	Cured (NR)
Papanikolaou et al^18^	Right costal trunk	*A. radingae*	*Actinotignum* spp., *Peptostreptococcus* spp., *Prevotella* spp.*, Porphyromonas* spp.*, Campylobacter* spp.	Empiric: amoxicillin/clavulanate and clindamycin Definitive: amoxicillin/clavulanate Oral: amoxicillin/clavulanate	IV: none; oral: 2 weeks	Cured (5 months)
Manasia et al^19^	Neck, mediastinum, anterior chest	*A. meyeri*	α-hemolytic *Streptococcus* spp.	Empiric: vancomycin and imipenem/cilastatin Definitive: NR Oral: NR	NR	Died
Sebastian et al^20^	Forearm	*A. odontolyticus*	*Streptococcus constellatus*, *Gemella* spp.	Empiric: piperacillin/tazobactam and linezolid Definitive: ampicillin/sulbactam Oral: amoxicillin/clavulanate 875 mg twice daily	IV: NR; oral: 6-12 months	Cured (NR)
Ventura et al^21^	Fournier’s gangrene	NR	*Candida albicans*	Empiric: vancomycin and meropenem Definitive: ampicillin/sulbactam and micafungin Oral: NA	IV: 14 days; oral: none	Cured (NR)
Lee et al^22^	Labia	NR	*Streptococcus viridans*, *Staphylococcus aureus*, *Enterococcus faecalis*, *Corynebacterium* spp.	Empiric: piperacillin/tazobactam, vancomycin, and clindamycin Definitive: NR Oral: amoxicillin/clavulanate	IV: NR; oral: 9-12 months	Cured (NR)
Tamesis et al^23^	Right iliopsoas muscle	*A. odontolytica*	*Escherichia coli*	Empiric: meropenem, vancomycin, and clindamycin Definitive: ampicillin/sulbactam Oral: NR	IV: 6 weeks; oral: NR	Cured (NR)
Contreras et al^24^	Left shoulder/left chest wall	*A. meyeri*	None	Empiric: aztreonam and vancomycin Definitive: ceftriaxone and clindamycin Oral: NR	NR	NR
Panwar et al^25^	Right medial thigh	*A. turicensis*	Unidentified anaerobic gram-negative organisms	Empiric: vancomycin and piperacillin/tazobactam Definitive: NR Oral: NR	NR	Cured (NR)
Gatti et al^26^	Abdominal wall, pelvic region, right thigh	*A. turicensis*	None	Empiric: piperacillin/tazobactam 4.5 g 4 times daily, daptomycin 8 mg/kg/day, and rifampicin 900 mg/day Definitive: ampicillin 12 g/day via continuous infusion; eventually increased to 16 g/day Oral: NA	IV: 5 weeks; oral: none	Cured (5 months)

Abbreviations: IV, intravenous; NA, not applicable; NR, not reported.

For many years, biochemical identification systems were the standard method used in clinical microbiology laboratories for bacterial identification. The ability to routinely identify *Actinomyces* and *Clostridium* species using these systems was limited. When matrix-assisted laser desorption ionization coupled with time of flight (MALDI-TOF) mass spectrometry was adapted for use in clinical microbiology laboratories, it provided a more robust method for accurate microbial identification. Before the advent of MALDI-TOF mass spectrometry, gram-positive bacilli such as these were primarily identified as diphtheroids or anaerobic diphtheroids.

When susceptibility testing is performed for uncommon organisms, such as *A. europaeus* and *C. innocuum*, results are difficult to interpret given the lack of consensus on what breakpoint values to use. For many *Actinomyces* species, including *A. europaeus*, applying the Clinical and Laboratory Standards Institute (CLSI) M45 *Corynebacterium* breakpoints is suggested.^27^ Until 2021, the European Committee on Antimicrobial Susceptibility Testing (EUCAST) published susceptibility breakpoints for gram-positive anaerobes, which included *Actinomyces* species. When the document was updated in 2022, the all-encompassing section on gram-positive anaerobes was removed. *Actinomyces* breakpoints are now found in a supporting document titled “When there are no breakpoints in breakpoint tables.”^28^ The new guidance document supports use of much lower breakpoints, especially for β-lactam antibiotics (Table 3). In contrast, breakpoints for β-lactams, which are considered the drug of choice, are more stringent in the CLSI document. For minimum inhibitory concentration (MIC) interpretation in *C. innocuum*, the CLSI M100 guidelines for anaerobic bacteria or EUCAST *C. perfringens* sensitivities should be used.^34,35^

**Table 3.** Minimum Inhibitory Concentration Breakpoints

Drug	*Actinomycin* species			*Actinomyces* + *Clostridium* species	*Clostridium* species	
	CLSI^29^	EUCAST^30^	CLSI^31,a^	EUCAST^28^	CLSI^32^	EUCAST^33^
Penicillin	NA	≤0.25 mg/L	≤0.12 mg/L	≤0.5 mg/L	≤0.5 mg/L	≤0.5 mg/L
Ampicillin or amoxicillin	NA	≤4 mg/L	NA	≤0.5 mg/L	≤0.5 mg/L	≤0.25 mg/L
Ampicillin/sulbactam or amoxicillin/clavulanate	≤8/4 mg/L	≤4/2 mg/L	NA	≤0.5 mg/L	8/4 mg/L	≤0.25 mg/L
Piperacillin or piperacillin/tazobactam	NA	≤8 mg/L	NA	≤2 mg/L	≤16/4 mg/L	≤0.5 mg/L
Ceftriaxone	≤8 mg/L	NA	≤1 mg/L	NA	≤16 mg/L	NA
Cefotaxime	NA	NA	≤1 mg/L	NA	NA	NA
Cefepime	NA	NA	≤1 mg/L	NA	NA	NA
Ertapenem	NA	≤0.5 mg/L	NA	≤0.25 mg/L	≤4 mg/L	≤0.5 mg/L
Meropenem	NA	≤2 mg/L	≤0.25 mg/L	≤1 mg/L	≤4 mg/L	≤0.5 mg/L
Imipenem	≤4 mg/L	≤2 mg/L	NA	≤1 mg/L	≤4 mg/L	≤0.5 mg/L
Vancomycin	≤2 mg/L	≤2 mg/L	≤2 mg/L	≤2 mg/L	≤8* mg/L	≤2 mg/L
Daptomycin	NA	NA	≤1 mg/L	NA	NA	NA
Linezolid	≤8 mg/L	NA	≤2 mg/L	≤2 mg/L	NA	NA
Doxycycline	≤1 mg/L	NA	≤4 mg/L	NA	NA	NA
Tetracycline	NA	NA	≤4 mg/L	NA	≤4 mg/L	NA
Minocycline	≤1 mg/L	NA	NA	NA	NA	NA
Ciprofloxacin	≤1 mg/L	NA	≤1 mg/L	NA	NA	NA
Moxifloxacin	≤1 mg/L	NA	NA	≤1 mg/L	≤2 mg/L	NA
Erythromycin	NA	NA	≤0.5 mg/L	NA	NA	NA
Clindamycin	≤2 mg/L	≤4 mg/L	≤0.5 mg/L	≤0.5 mg/L	≤2 mg/L	≤0.25 mg/L
Trimethoprim/sulfamethoxazole	≤2/38 mg/L	NA	≤2/38 mg/L	NA	NA	NA
Rifampin	≤1 mg/L	NA	≤1 mg/L	≤0.125 mg/L	NA	NA
Metronidazole	NA	≤4 mg/L	NA	≤4 mg/L	≤8 mg/L	≤4 mg/L
Amikacin	≤8 mg/L	NA	NA	NA	NA	NA
Gentamicin	NA	NA	≤4 mg/L	NA	NA	NA
Tobramycin	≤4 mg/L	NA	NA	NA	NA	NA

Abbreviations: CLSI, Clinical and Laboratory Standards Institute; EUCAST, European Committee of Antimicrobial Susceptibility Testing; NA, not applicable.

^a^It is recommended to use *Corynebacterium* susceptibility breakpoints for aerotolerant strains of *Actinomyces* (*Actinomyces bovis*, *Actinomyces europaeus*, *Actinomyces graevenitzii*, *Actinomyces johnsonii*, *Actinomyces naeslundii*, *Actinomyces neuii*, *Actinomyces odontolyticus*, *Actinomyces oris* group, *Actinomyces radingae*, *Actinomyces turicensis*, *Actinomyces urogenitalis*, *Actinomyces viscosus*). This is also recommended for *Actinomyces dentalis* and *Actinomyces israelii*.

In our case, *Actinomyces*, *C. innocuum*, and *Bacteroides* were identified via the MALDI-TOF methodology. Most clinical microbiology laboratories do not routinely report species identification or perform susceptibility testing; however, species identification for both *Actinomyces* and *Clostridium* is imperative because it helps guide appropriate empiric therapy, especially in a life-threatening infection, because variation in susceptibility patterns between species exist. In this case, susceptibility testing was ordered for all organisms, but results were available on day 15 for only *C. innocuum* and *B. fragilis*. Delays associated with use of a reference laboratory made it difficult to optimize antimicrobial therapy. Our *C. innocuum* isolate was sensitive to all antibiotics tested via CLSI criteria but resistant to clindamycin, metronidazole, meropenem, and piperacillin/tazobactam according to EUCAST criteria. Interpretation is difficult given the variability in results; this could have contributed to the patient requiring numerous washouts in the operating room while on therapy.

If susceptibility testing is not readily available or CLSI guidelines are not available for interpretation of susceptibility, practitioners are forced to rely on the literature to determine appropriate therapy. Treatment of actinomycosis is based on the species isolated and whether the infection is monomicrobial or polymicrobial. In previous literature, penicillin G, penicillin V, amoxicillin, and ampicillin are considered first-line agents for monomicrobial infections.^3^ However, not all agents in the penicillin class have the same in vitro activity. *Actinomyces* species are intrinsically resistant to penicillinase-resistant penicillins such as oxacillin, nafcillin, and dicloxacillin. In susceptibility testing by E-test (bioMerieux, Mercy-l’Étoile, France) methodology using CLSI breakpoints for over 500 *Actinomyces* isolates (*A. odontolyticus*, 24% of isolates; *A. oris*, 19% of isolates; the species was not identified for 58% of isolates), a penicillin MIC range of 0.002 to 4 mg/L was reported, with 97.3% of isolates reported to be susceptible to penicillin.^36,37^ Although *Actinomyces* species are not known to produce β-lactamase, concomitant organisms in polymicrobial infections may produce β-lactamases requiring the use of a β-lactamase inhibitor such as ampicillin/sulbactam, amoxicillin/clavulanate, or piperacillin/tazobactam.

Cephalosporins also have activity against *Actinomyces* species; however, the literature recommends avoiding first-generation cephalosporins for empiric treatment.^3^ Steininger et al^38^ reported in vitro sensitivity to cefazolin for isolates of *A. meyeri* and *A. radingae*, although clinical data are lacking. Of the cephalosporins, cefoxitin and ceftriaxone are active in vitro and have been used in clinical practice.^39^ Certain *Actinomyces* species are sensitive to cephalothin, cefuroxime, and cefotaxime, but all are considered less potent than penicillin.^40^ Ceftazidime has poor in vitro activity. Carbapenems are also active in vitro and have been used in clinical practice.^41^

Non–β-lactam antimicrobials with activity against *Actinomyces* species include linezolid, vancomycin, daptomycin, tetracyclines, glycylcyclines, lincosamides, and macrolides. Vancomycin is generally 2- to 4-fold more active than daptomycin against *Actinomyces* species.^42^  *Actinomyces* species inactivate daptomycin by hydrolytic ring opening or diacylation of daptomycin’s lipid tail, resulting in levels of resistance of up to 80%.^43^ In patients with true severe penicillin allergies who cannot tolerate extended-spectrum cephalosporins, doxycycline is recommended. Clindamycin also has activity, but the sensitivity ranges from 33% to 82% depending on the species.^38^  *Actinomyces* species are intrinsically resistant to metronidazole and trimethoprim/sulfamethoxazole. Fluoroquinolones have activity but are not efficacious.

Four cases of *A. europaeus* necrotizing fasciitis have been reported, with a clinical cure achieved in only 2 cases; definitive therapy was described in only 1 case, consisting of linezolid and ciprofloxacin followed by meropenem, metronidazole, and linezolid due to clinical worsening.^10,12^ Smith et al^37^ reported a 20% resistance rate to piperacillin/tazobactam for *A. europaeus* with an overall higher MIC range (0.047 to 32 mg/L) compared to other *Actinomyces* species. Anthony et al^11^ reported a case of piperacillin/tazobactam failure believed to be related to resistance in a case of *A. europaeus* necrotizing fasciitis. Resistance to ceftriaxone and linezolid has also been reported in *A. europaeus* isolates.^37^

Compared to *C. perfringens*, which is typically sensitive to β-lactams, clindamycin, metronidazole, and vancomycin, *C. innocuum* has been reported to be resistant to β-lactams and clindamycin. Resistance rates to clindamycin and penicillin range from 4.1% to 68.2% and from 12.5% to 79.5%, respectively.^6,44^ Unlike other *Clostridium* species, *C. innocuum* is intrinsically resistant to vancomycin.^45^ The MIC_90_ reported included amoxicillin/clavulanate and ampicillin/sulbactam at 0.5 mg/L, clindamycin and metronidazole at 1 mg/L, meropenem and piperacillin/tazobactam at 2 mg/L, penicillin and vancomycin at 8 mg/L, and cefoxitin at 128 mg/L.^5,42^ Metronidazole and ampicillin/sulbactam are typically active against this organism. Linezolid and daptomycin MICs have been reported as 2 to 4 mg/L and 2 to 8 mg/L, respectively.^46^ Given that the MIC values for most antimicrobial agents vary widely for *C. innocuum*, treatment is dependent on results from susceptibility testing.

Treatment duration for necrotizing infections that involve *Actinomyces* species must be individualized to the patient, as there are no evidence-based guidelines. The 2014 guidelines on skin and soft tissue infections from the Infectious Diseases Society of America do not include treatment recommendations for *Actinomyces* infections.^47^ They state that necrotizing infections require antibiotic therapy until no further surgical intervention is needed, the patient improves clinically, and fever abates for 48 to 72 hours, but these recommendations may not be appropriate for patients with *Actinomyces* infections. Duration of therapy ranges from just 7 days to 12 months, varying widely given the limited literature and risk of recurrence. For necrotizing fasciitis, the total duration of therapy reported in cases with a successful outcome was 14 days (n = 2), 21 days (n = 2), 35 days (n = 1), 42 days (n = 2), and 6 to 12 months (n = 2).^12,15,17,18,20-23,26^ It is difficult to determine true success, because case reports had various times to follow-up. Some authors have hypothesized that duration of therapy may be related to the *Actinomyces* species. One recommendation is to treat *A. israelii*, *Actinomyces gerencseriae*, *A meyeri*, *A. odontolyticus*, and *A. viscosus*/*naeslundii* with 2 to 6 weeks of IV penicillin followed by 6 to 12 weeks of oral penicillin or amoxicillin.^48^ A 3-month regimen may be appropriate for certain species such as *A. europaeus*, *Actinomyces funkei*, *Actinomyces neuii*, *A. radingae*, and *A. turicensis*. Other authors have stated that the duration of therapy is related to source control with the removal of all infected tissue, which may be linked to shorter treatment courses for necrotizing fasciitis.^12,18,21^ For our patient, consideration was given to converting to an oral antimicrobial regimen, once clinically appropriate, to complete a total duration of 3 months of therapy. Given that the literature was both extremely limited and variable, as well as the high risk of recurrence, we felt that this duration would be appropriate because of the severity of the infection.

## Conclusion

Necrotizing fasciitis caused by *A. europaeus* and *C. innocuum* is rare. A common empiric regimen for necrotizing fasciitis is IV vancomycin, piperacillin/tazobactam, and clindamycin, but the regimen may not be optimal for these pathogens. It is important to rapidly identify the species for both *Actinomyces* and *Clostridium* and obtain sensitivity data to help ensure proper therapy is given, but sensitivity date are difficult to obtain quickly when testing is preformed by a reference laboratory. Interpretation of susceptibility results is not currently available, so practitioners need to review both CLSI and EUCAST recommendations for a complete antimicrobial susceptibility profile as CLSI and EUCAST report different MIC breakpoints for the same organism. For both organisms, ampicillin or ampicillin/sulbactam is appropriate therapy. For *Actinomyces* infections, the duration of therapy should be individualized and can range from 14 days to 1 year based on patient response and the species identified.

## Data Availability

The data underlying this article are available in the article.
